# The Crystal Structure and Morphology of 2,4,6,8,10,12-Hexanitro-2,4,6,8,10,12-hexaazaisowurtzitane (CL-20) *p*-Xylene Solvate: A Joint Experimental and Simulation Study

**DOI:** 10.3390/molecules191118574

**Published:** 2014-11-13

**Authors:** Fanfan Shen, Penghao Lv, Chenghui Sun, Rubo Zhang, Siping Pang

**Affiliations:** 1 School of Life Science, Beijing Institute of Technology, Beijing 100081, China; E-Mail: shenfanfan666@163.com; 2 School of Material Science & Engineering, Beijing Institute of Technology, Beijing 100081, China; E-Mail: lv2120121236@163.com; 3 School of Chemistry, Beijing Institute of Technology, Beijing 100081, China; E-Mail: zhangrubo@bit.edu.cn

**Keywords:** crystal morphology, molecular dynamic simulation, solvent effect, CL-20, *p*-xylene

## Abstract

The crystal structure of 2,4,6,8,10,12-hexanitro-2,4,6,8,10,12-hexaazaiso-wurtzitane (CL-20) *p*-xylene solvate, and the solvent effects on the crystal faces of CL-20 were studied through a combined experimental and theoretical method. The properties were analyzed by thermogravimetry-differential scanning calorimetry (TG-DSC), Fourier transform infrared spectroscopy (FTIR) and X-ray diffraction (XRD).The growth morphology of CL-20*p*-xylene solvate crystal was predicted with a modified attachment energy model. The crystal structure of CL-20*p*-xylene solvate belonged to the *Pbca* space group with the unit cell parameters, *a* = 8.0704(12) Å, *b*=13.4095(20) Å, *c* = 33.0817(49) Å, and *Z* = 4, which indicated that the *p*-xylene solvent molecules could enter the crystal lattice of CL-20 and thus the CL-20 *p*-xylene solvate is formed. According to the solvent-effected attachment energy calculations, (002) and (11−1) faces should not be visible at all, while the percentage area of the (011) face could be increased from 7.81% in vacuum to 12.51% in *p*-xylene solution. The predicted results from the modified attachment energy model agreed very well with the observed morphology of crystals grown from *p*-xylene solution.

## 1. Introduction

Crystal morphology is very important because it can determine many properties of a material, such as shelf life, vapor pressure, solubility, bioavailability, and density, which further influence the related downstream products [1]. For example, the crystal morphology of explosives is one of their most significant properties and can decide their safety characteristics [2]. With the same size, needle-shaped explosive crystals are more mechanically and thermally sensitive than spherical ones [3]. During the crystal growth, crystal morphology is usually regulated by two factors, the internal structure of crystal and its external parameters, such as supersaturation, temperature, solvent and even impurities. Solvent effects on the crystal morphology of explosives [4,5] and pharmaceuticals [6] have been well studied theoretically [7,8,9,10,11,12] with the Bravais-Friedel-Donnay-Harker (BFDH) model [13,14], the attachment energy (AE) model [15] and the Burton-Cabrera-Frank (BCF) model [16], showing clearly that influence of solvent-surface interaction energy can be used to predict crystal morphology in solvents. As the most appropriate model among those established methods, AE is calculated for a series of suitable slices (*h k l*) that are chosen by performing a Donnay-Harker prediction. From the energy calculation, and hence the growth rate, a center-to-plane distance is assigned to each face. This information is used to deduce the morphology. The modified AE model is successfully applied to the prediction of crystal morphology and verified experimentally [17,18].

The caged 2,4,6,8,10,12-hexanitro-2,4,6,8,10,12-hexaazaisowurtzitane(CL-20), first synthesized by Nielsen [19], with a rigid isowurtzitane cage and nitro groups attached to the bridging nitrogen atoms, is currently the most powerful commercially available explosive. The spatial orientations of these nitro groups with the respect to the five-member and six-member rings in the isowurtzitane cage, together with the crystal packing, give rise to rich polymorphism, which has been explored in detail previously [20,21,22]. For instance, CL-20 can be recrystallized into four polymorphic forms, including alpha (α),(which is a hydrate) and non-solvated crystal forms (β, γ, ε), under ambient conditions in solvent and mixed solvents [23]. Crystal structures of CL-20 solvates, such as N,N-dimethylformamide (DMF) solvate, 1,4-dioxane solvate, hexamethyl phosphoramide (HMPA) solvate and butyrolactone solvate were studied previously [24], but little work has been focused on both the CL-20 crystal morphology of different solvates and the quantitative solvent effects. Since the explosiveCL-20 crystallizes in the solvent [25], it is very important to study the solvent effects on CL-20 crystal faces and morphology.

In this present work, a combination of experimental and simulated morphology had been done to allow us to understand roles of solvents on crystal morphology. The crystal transformation of CL-20 was performed in ethyl acetate-*p*-xylene solution, and CL-20 *p*-xylene solvate was obtained. Molecular dynamic (MD) simulation was used to predict the interaction strength between *p*-xylene solvent molecules and CL-20 crystal faces, and the crystal morphology of CL-20 *p*-xylene solvate. All of the predicted morphologies were consistent with the experimental results, which validated the growth model of explosive CL-20 crystals in *p*-xylene solution at a given temperature. These results provided the fundamental information to predict the potential morphology changes during the formation of solvated CL-20 or other explosives.

## 2. Computational Theoryand Details

### 2.1. Computational Theory

The growth morphology algorithm is based on the attachment energy (AE) method, which is proposed by Hartman and Bennema based upon period bond chain (PBC) theory [26]. The attachment energy, E_att_, is defined as the released energy of a growth slice to a growing crystal surface. E_att_ is computed as [27]:

E_att_ = E_latt_ − E_slice_ (1)

where E_latt_ is the lattice energy of the crystal, E_slice_ is the energy of a growth slice of thickness d_hkl_, the relative growth rate in vacuum of the crystal surface, R_hkl_, is assumed to the proportional to the attachment energy:

R*_hkl_* ∞ │E*_att_*│ (2)

The modified morphology is generated based on the relation stated by Hartman and Bennema, where R'_hkl_ represents the growth rate in a particular direction, which is directly proportional to the modified attachment energy [28]:

R'*_hkl_* ∞ │^mod^E*_att_*│ (3)

The solvent can be assumed to reduce the growth rate. First, the solvent needs to be removed from the surface before the crystal face can grow, which costs energy and herewith the apparent attachment energy decrease. So modified AE can be introduced with the following formula:

^mod^E_att_ = E_att_ − SE_s_ (4)

where correction factor S reflecting the surface features and the effect of solvent volume, defined as the accessible solvent surface per unit crystal area [29]:

(5) $$\text{S}=\frac{{\text{A}}_{\text{acc}}}{{\text{A}}_{\text{hkl}}}$$

where A_acc_ is the area of accessible solvent surface of an (*h k l*) slice in the unit cell, A_hkl_ is the surface area of an (*h k l*) slice in the unit cell.

Since the effect of solvent depends on both the different surface chemistry and the associated topography (step structures) of the crystal face, the correction term E_s_ describing the energy of solvent binding on the crystal surface (*h k l*) can be calculated using the following formula [30]:

(6) $${\text{E}}_{\text{S}}=\frac{{\text{E}}_{\text{int}}{\text{A}}_{\text{acc}}}{\text{N}{\text{A}}_{\text{model}}}$$

where E_int_ is the interaction energy between the solvent layer and the surface, A_acc_ is the area of accessible solvent surface of an (*h k l*) slice in the unit cell, A_model_is the total surface area of modeling box in the (*h k l*) direction. N is the number of CL-20 in the crystal layer. E_int_is the interaction energy between the solvent layer and the surface, which is evaluated using the relationship:

E_int_ = E_total_ − E_surf_ − E_solv_ (7)

where E_total_ is the total energy of the surface and the solvent layer, E_surf_ is the energy of the surface without the solvent layer, and E_solv_ is the energy of solvent layer without the surface.

### 2.2. Computational Details

**Figure 1:**
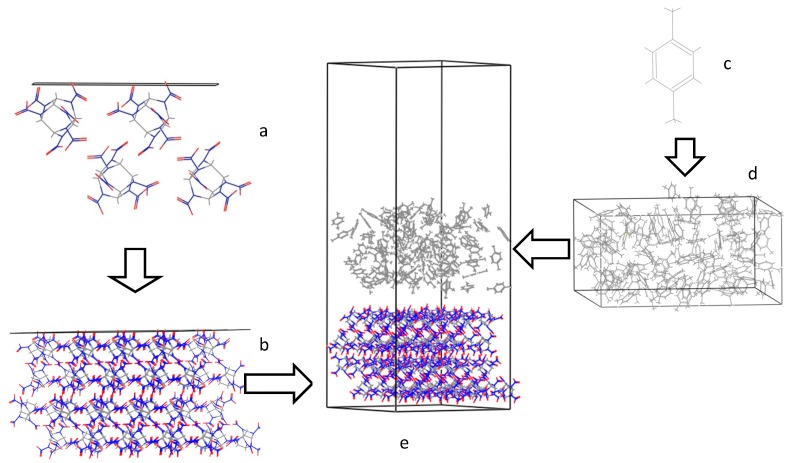
The schematic representation of CL-20 surface and *p*-xylene solvent interfacial model (**a**) crystal face from unit cell; (**b**) crystal layer (30 × 30 Å^2^); (**c**) *p*-xylene molecule; (**d**) solvent layer (solvent bulk of *p*-xylene); and (**e**) modeling box (crystal layer + solvent layer + vacuum slab).

All of the MD and morphology predictions were done with the commercial molecular modeling software package Materials Studio 5.5 [31] with the Compass force-field. The crystal structure of γ-CL-20 (CCDC number 117,778) [32] was optimized with the forcite algorithm. Subsequently, the crystal morphology of CL-20 in vacuum was predicted with AE model to acquire the main stable crystal faces withdifferent miller indices (*h k l*).Then, the CL-20 crystal was cleaved along the main stable faces (10−1), (101), (011), (002), (110) and (11−1) with a depth of 2d_hkl_. The corresponding crystal layer was built as a periodic superstructure of about 30 × 30 Å^2^ unit cell. A solvent layer filled with 100 random distributed p-xylene molecules was constructed with the amorphous cell tool with a layer density of 0.8611g/cm^3^and whose size was set appropriate to the crystal layer. The two-layer interfacial model was employed to investigate solvent effect on the crystal morphology (Figure 1).

In this model, the two layers were a crystal slice and asolvent layer, *i.e.*, the solvent layer docked CL-20 crystal surface along c axis and moved freely in the simulation. And a vacuum slab thickness of 30 Å was built above the solvent layer to eliminate the effect of additional free boundaries on the structure. Energy minimization for the interfacial model was carried out before the dynamics simulation. MD simulation was performed for 500 ps with a time step of 1 fs (T = 298 K and P = 1 atm) with the NVT ensemble [33].Coupling to the heating bath was carried out with the Andersen thermostat [34]. After the system was equilibrated at the target temperature, the interfacial configurations were sampled for 100 ps with a sampling interval of 1 ps. The Compass force-field, a powerful *ab initio* force-field parameterized and validated using condensed phase properties, enabled the accurate and simultaneous prediction of structural, conformational, vibrational, and other physical properties for a broad range of covalent molecules including most common organics [35]. For potential-energy calculations, the Coulomb interactions and Van der Waals forces were calculated by employing the standard Ewald method with a calculation accuracy of 0.001kcal/mol and cutoff distance was set as 12.5 Å [36,37].

## 3. Results and Discussion

### 3.1. Crystal Structure

Single crystal X-ray diffraction showed that CL-20 *p*-xylene solvate crystal belonged to the orthorhombic system, space group*Pbca*, with the unit cell parameters, *a* = 8.0704(12) Å, *b* = 13.4095(20) Å, *c* = 33.0817(49) Å, *V* = 3580.10(92) Å^3^, and *D_crystal_* = 1.8228 g/cm^3^.The measured molecular weight of CL-20p-xylene solvated crystal was 3930.16 g/mol, which was equal to the total molecular weight summation of CL-20 andp-xylene, indicating that the molecular number ratio of CL-20 to*p*-xylene was 1:1. Additionally, the molecular ratio of CL-20 to *p*-xylene could be founddirectly from theunit cell.Crystallographic data and structure refinement parameters for CL-20 *p*-xylene solvate are listed in Table 1.

**Figure 2:**
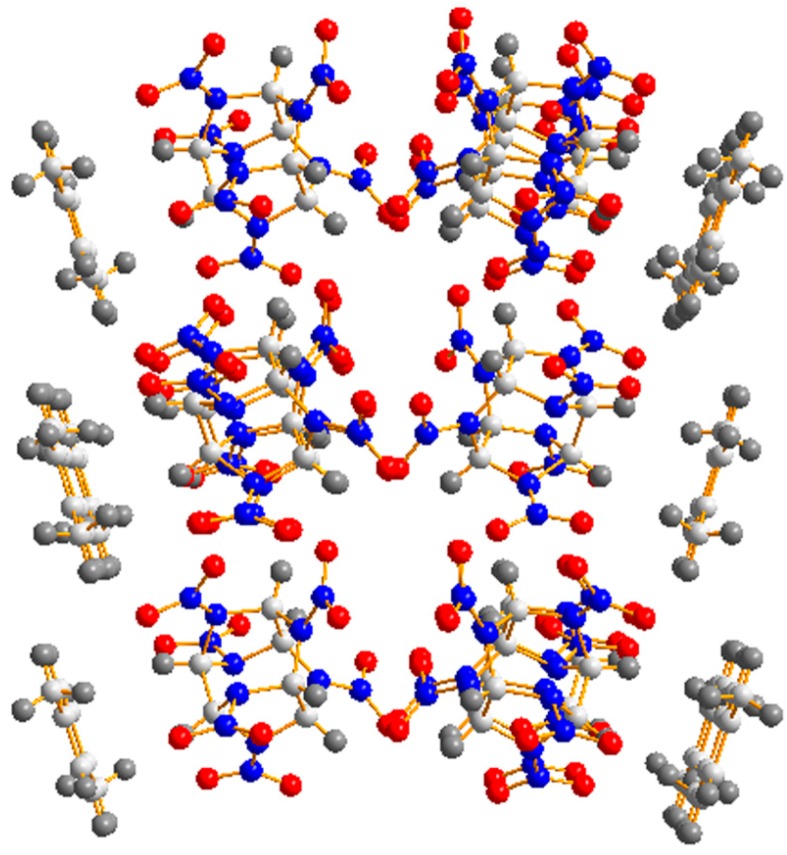
The layered arrangement observed in CL-20 *p*-xylene solvate (view along a-axis).

In the CL-20 *p*-xylene solvate crystal packing, the γ-conformation of the CL-20 molecules was observed in the crystal structure of the solvate, in which there was one *p*-xylene molecule for every CL-20 molecule. Alternation of two CL-20 layers and one *p*-xylene layer could be found, as shown in Figure 2. In the CL-20 layers, individual molecules with both the molecular conformation and the relative spatial distribution observed in the γ-polymorph of CL-20 crystals (as shown in Figure 2). The C–H…O intermolecular interactions resulted in a network of weakly linked CL-20 molecules along two dimensions. The “face-to-face” arrangement (all six-membered rings of the cage) was oriented to c-axis, which leaded to the significative CL-20/CL-20 intermolecular interactions in the structure.

**Figure 3:**
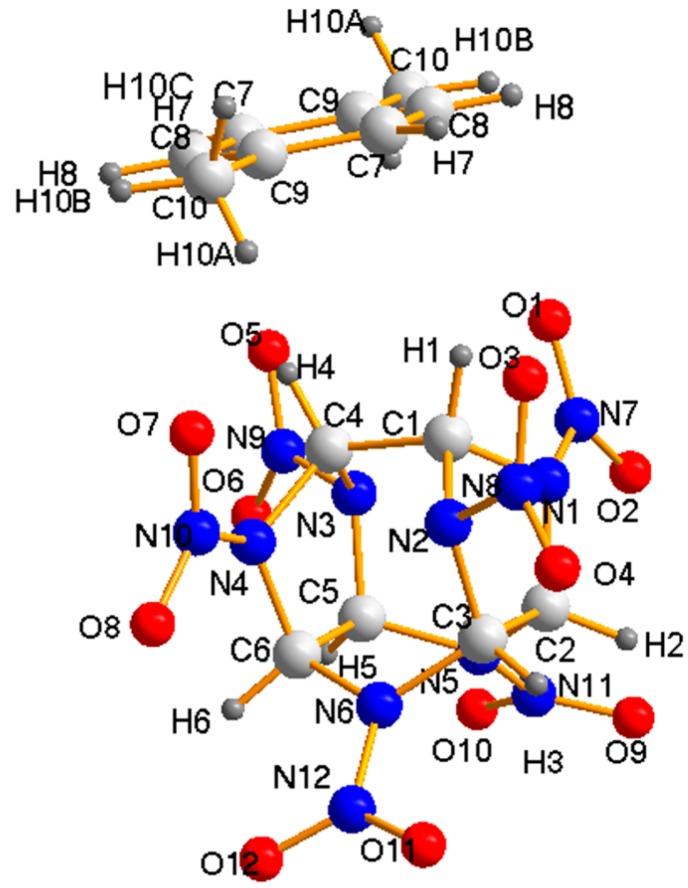
Diamond drawing of CL-20 *p*-xylene solvate showing the atom-labeling scheme and molecular conformation.

In the crystal structure, the most important intermolecular interactions between the hydrogen atoms (H) of the *p*-xylene molecules and the NO_2_ groups of CL-20 molecules were the primary driving force for the formation of CL-20 *p*-xylene solvate, which also made contribution to the stability of the crystal structure (Figure 3). The possible hydrogen bond distances and angles are listed in Table 2 with the labeled atoms in Figure 3. In addition to the intermolecular hydrogen bond interactions, the O…C, and O…N stacking interactions between CL-20 and *p*-xylene played an important role in the stabilization of the solvated structure, which brought the two molecules together to form the CL-20 *p*-xylene solvate (Table 1). For example, the very strong C10-H10A…O5 hydrogen bond between the hydrogen atom of *p*-xylene and the nitro group of CL-20 was one of the main forces to form CL-20 *p*-xylene solvate, which had a bond distance of 2.6995 Å and bond angle of 166.6°.

**Table 1:** Crystallographic data and structure refinement parametersfor CL-20 *p*-xylene solvate.

CCDC	1022276
Empirical formula	C_20_H_22_N_24_O_24_
Temperature (K)	153(2)
Wavelength (Å)	0.71073 Å
Space group	Pbca
Z	4
a (Å)	8.0704(12)
b (Å)	13.410(2)
c (Å)	33.082(5)
ɑ(°)	90
β(°)	90
γ(°)	90
Volume (Å^3^)	3580.1(9)
Density(calculated) (g cm^−3^)	1.823
µ (mm^−1^)	0.167
F(000)	2008
Crystal size (mm)	0.44 × 0.38 × 0.21
*θ*(°)	2.81–29.13
Limiting indices	−11 ≤ h ≤ 11 −14 ≤ k ≤ 28 −41 ≤ l ≤ 45
Reflections Collected	4814
Independent reflections	4589
Final R indices [I > 2σ(I)]	R_1_ = 0.0528 ωR_2_ = 0.1488
Final R indices (all data)	R_1_ = 0.0566 ωR_2_ = 0.1528

**Table 2:** Intermolecular interactions between CL-20 and *p*-xylene.

D–H…A	D–H (Å)	H–A (Å)	<DHA (deg)
**Hydrogen Bonds**			
C7H7…O1	0.9501(1)	2.8122(3)	127.893(3)
C7H7…O3	0.9501(1)	2.9562(3)	106.225(7)
C8H8…O3	0.9499(1)	2.8723(3)	148.665(8)
C10H10A…O5	0.9800(3)	2.7012(2)	166.622(8)
C10H10B…O3	0.9800(1)	2.7530(3)	155.673(8)
C10H10B…O1	0.9800(1)	2.9322(4)	96.873(7)
C10H10C…O5	0.9799(1)	2.7267(3)	141.969(8)
C10H10C…O1	0.9799(1)	2.8850(3)	99.801(7)
**Stacking Interactions (Å)**			
O1…C7	3.4775(4)	O1…C8	3.5569(3)
O1…C9	3.4060(4)	O1…C10	3.2009(3)
O3…C7	3.3484(3)	O5…C7	3.4079(3)
O7…C8	3.2757(4)	O7…C9	3.2217(5)
N7…C7	3.9333(4)	N7…C8	4.0854(4)

### 3.2. Solvate Properties

**Figure 4:**
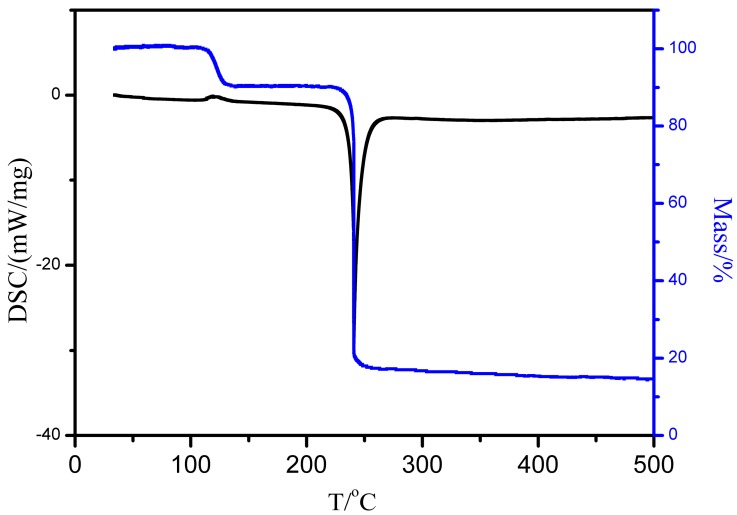
Thermograms of CL-20-*p*-xylene solvate (dried at room temperature).

The TG-DSC curve of CL-20-*p*-xylene solvate (dried at room temperature) is presented in Figure 4. There is a gradual weight loss around 105 °C due to the desolvation and a steep weight loss from 225 to 240.8 °C owing to decomposition. Two peaks were found on the DSC curve. The first peak at 105 °C was attributed to desolvation, in conformity with the slight weight loss. The second sharp peak at 240.8 °C could be assigned to the CL-20 decomposition [38].

**Figure 5:**
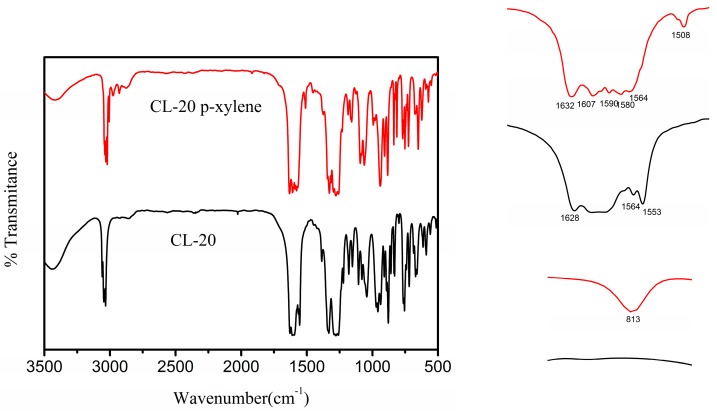
FTIR of selected CL-20 formulations, where the red line represents the CL-20 *p*-xylene solvate, and the black line represents the γ-CL-20.

FTIR spectroscopy is normally used for the phase determination of CL-20 and works very well in determination of the predominant phase and the changes caused by intermolecular hydrogen bonds are relatively apparent in the infrared spectrum [39]. The FTIR spectrum showed that several regions were highly sensitive to the structure changes of CL-20 *p*-xylene solvate (Figure 5).

The 813 cm^−1^ band could be assigned to C-H flexural out-plane vibration of *p*-xylene. Bands at 1600 cm^−1^, 1580 cm^−1^ and 1500 cm^−1^due to C=C stretching of benzene ring appear in the CL-20 *p*-xylene solvate structure. Among the three nitramine group stretching bands of CL-20, including ^as^(NO_2_) (antisymmetric stretching vibration),^s^(NO_2_) (symmetric stretching vibration) and (N-N), only ^as^(NO_2_) could be considered as a characteristic one to study [40]. In CL-20 *p*-xylene solvate, the 1628–1553 cm^−1^bands could be assigned to asymmetrical NO_2_ stretching of CL-20, which was shifted to 1632–1564 cm^−1^ due to the interactions between CL-20 and *p*-xylene. The symmetrical NO_2_ stretching modes were mixed with other vibrations in the same region, especially with C-H bonds. Based on TG-DSC and FT-IR spectra, it could be confirmed that theCL-20*p*-xylene solvate was formed.

### 3.3. Crystal Morphology Prediction

**Figure 6:**
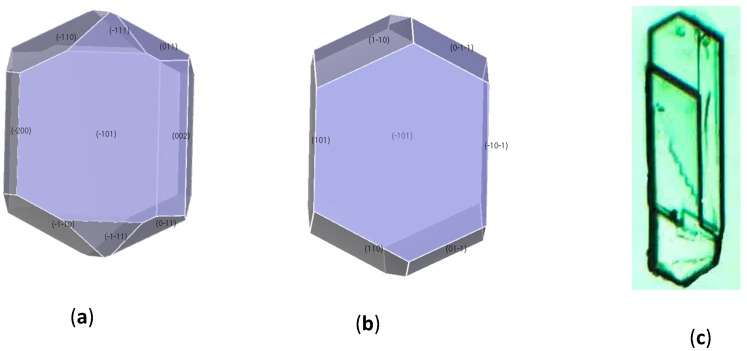
Predicted and experimental crystal morphologies of CL-20 grow from (**a**) vacuum (**b**) *p*-xylene (**c**) the crystal morphology observed by microscope.

The external morphology of crystals was predicted with the AE model. The predicted crystal morphology in vacuum was much different from the observed morphology in solvent as shown in Figure 6. The crystal habit in vacuum had an aspect ratio of 1.91. The unique exhibiting faces were (10−1), (101), (011), (002), (110) and (11−1), among which (10−1), (101) and (110) were the main faces with percentage area of 32.02%, 15.27%, and 23.00%, respectively.

**Figure 7:**
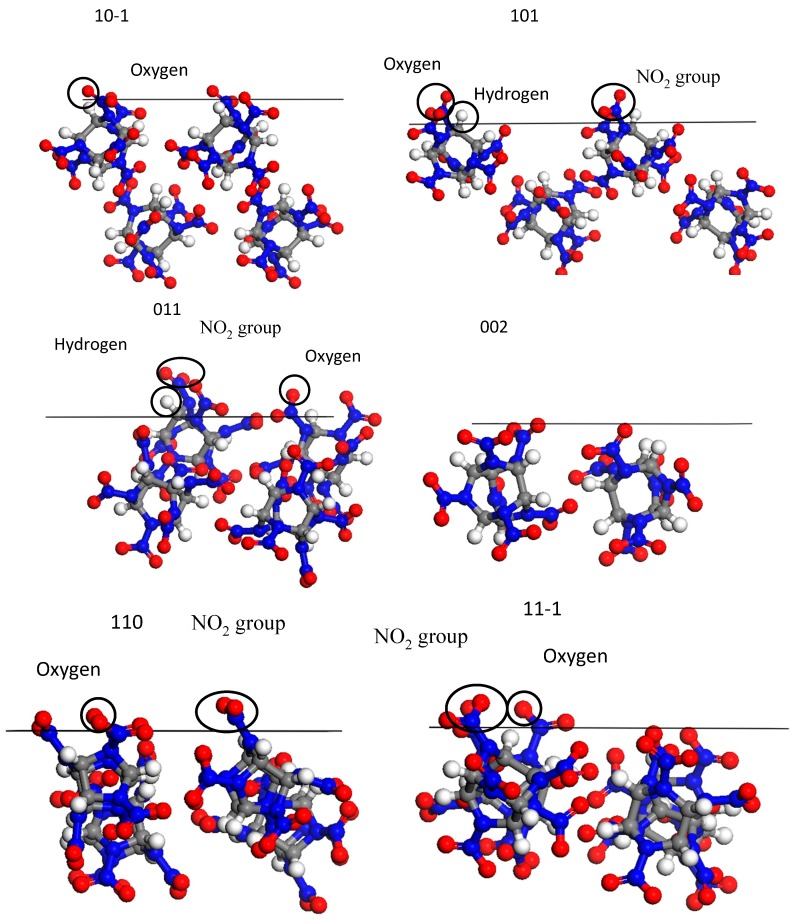
The molecular arrangements of (10−1), (101), (011), (002), (110) and (11−1) face of CL-20 crystal, the solid line represents the crystal face.

The crystal packing diagrams (Figure 7) revealed the surface chemistry and topography of CL-20 crystal morphology faces. The (101) and (011) faces were rather open and rough on the molecular level with nitro groups, hydrogen and oxygen atoms of the CL-20 exposed on the surface. Oxygen atoms were only observed exposed on the (10−1) face. The surface chemistry of (110) and (11−1) faces were mainly dominated by the exposed oxygen atoms and nitro groups. The (002) face was found to be smooth on the molecular level. Surface areas of six crystal faces were listed in Table 3. In the Connolly surface calculation, the grid interval was 0.4000 Å at a probe radius of 1.0 Å. The accessible solvent surfaces area of unit crystal denoted as S in Table 3. The A_acc_ and S were calculated quantitatively to compare the step structures of different crystal faces. As listed in Table 3, it was found that the (101) face had the highest S value, and the (002) face had the least S value on the contrary among all the exhibiting surfaces.

**Table 3:** Surface areas for the crystal habit surfaces.

Surface	(10−1)	(101)	(011)	(002)	(110)	(11−1)
A_acc_ (Å^2^)	381.7	600.1	694.4	264.9	624.28	642.6
A_model_ (Å^2^)	133.5	187.3	224.6	108.1	231.3	254.1
S	2.859	3.204	3.092	2.451	2.699	2.528

A_hkl_: the surface area of the crystal face (*h k l*) in unit cell; A_acc_: the accessible solvent surface of unit area.

The solvent effect E_s_ and modified attachment energy ^mod^E_att_ were calculated with formulas (1–6). The solvent effects depended on the different surface chemistry and the step structure of the crystal faces, which were quantified as the energy correction term E_s_ in this study. With a stronger interaction, it was found that the absolute value of the interaction energy was larger. For example, the low growth rate of (011) face in the *p*-xylene solution could be attributed to the polar nitro groups, hydrogen atoms and oxygen atoms exposed on the surface, and the large accessible solvent surface, which would be conducive to the easy solvent binding. Therefore, the desolvation process became very difficult with the high energy barrier, and the solute molecules were hampered to reach the surface. In the (002) face, its absolute value of the E_s_ was the lowest, resulting in the fastest growth in this direction. Inspite of the nearly equal interaction energies of (101) and (110) faces with the *p*-xylene molecules, the difference in the accessible solvent surface of unit area (see the S value in Table 2) had brought faster growth of (110) face than (101) face.

It is well known that the morphological importance of crystal faces is inversely proportional to their growth rates, which is affected significantly by the solvent. In a particular direction, the more negative the attachment energy is, the faster the growth rate will be and the less morphological importance the crystal face will have. As shown in Table 4, the attachment energies of all the habit faces were decreased with the solvent effect taken into consideration. This decrease indicated that interactions of the solvent molecules with the crystal layers were more favored compared to the CL-20 molecule itself. Therefore, the solvent effect restrained the growth rate. The comparison between Figure 6a,b revealed that the faster relative growth rates of (002) and (11−1) faces led to their disappearance in the final crystal habit. Conversely, the area percentage of (011) was increased up to 12.51% in *p*-xylene solution from 7.81% in vacuum due to strong solvent interactions. The changes of relative growth rates leaded to crystal shape which was close to a hexagon with the aspect ratio of 1.95, which was consistent with the observed morphology in *p*-xylene solvent. These results of the morphological predictions (Figure 6b) were in good agreement with the experimental morphology (Figure 6c), which indicated the successful prediction of the modified AE model.

**Table 4:** Calculated attachment energies for dominant crystal morphology faces together with corrected attachment energies and relative growth rates for main faces.

Face	d*_hkl_*	E_att_	R^c^	E_int_	A_acc_	A_model_	E_s_	^model^E_att_	R'_hkl_
(10−1)	11.38	−56.46	1	−100.874	381.7	534.09	−4.505737102	−43.5736	1
(101)	8.11	−78.10	1.38	−159.568	600.1	749.23	−7.987929119	−52.5087	1.21
(011)	7.06	−99.17	1.76	−168.444	694.41	871.47	−8.138949579	−73.9982	1.69
(002)	7.02	−83.54	1.48	−88.3024	264.93	432.39	−3.381490851	−75.2512	1.73
(110)	6.84	−87.61	1.55	−183.968	624.28	875.90	−7.757439759	−66.6756	1.53
(11−1)	6.64	−91.53	1.62	−159.496	642.56	1017	−6.501126945	−75.5951	1.73

### 3.4. Radial Distribution Functions of the Interfacial Model

**Figure 8:**
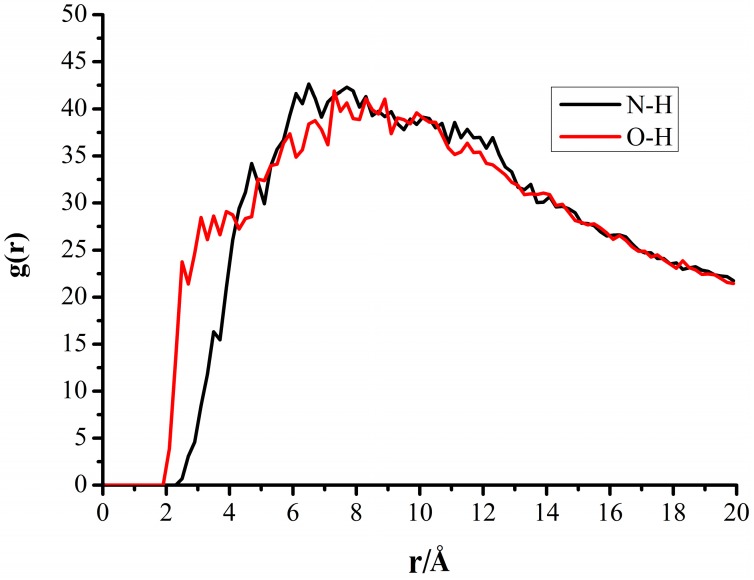
Radial Distribution Function of the interfacial model of *p*-xylene molecules and (10−1) face oftheCL-20.

The radial distribution function (RDF) gives a measure of the probability that, given the presence of an atom at the origin of an arbitrary reference frame, there would be an atom with its center in a spherical shell with infinite thickness and a distance, r, from the reference atom. In the radial function graph g(r)-r, the peaks were all within 3.0 Å, which indicated the hydrogen bonds between O atoms and H atoms [41]. Some high peaks existed in the region of 3.0–8.0 Å, implying the strong Van der Waals’ forces and the electrostatic interactions between this atom pair. [42] In order to analyze the solvent-crystal interactions clearly, the interface structures between CL-20 (10−1) faces and *p*-xylene molecules were explored with RDF(s), as shown in Figure 8.

The RDF(s) graphs of two pairs of atoms were plotted, which were hydrogen atoms in *p*-xylene molecules to oxygen atoms and nitrogen atoms of the nitro groups in CL-20 molecules, respectively denoted as H_p-xylene_-O_CL-20_ and H_p-xylene_-N_CL-20_ pairs. Similar RDF(s) could be obtained when analyzing other interfacial model systems comprising p-xylene molecules and the other five crystal faces.

The sharp peaks of O–H with r = 2.5 Å and 3 Å indicated the existing hydrogen bonds between the O and H atoms. With the interval of 3–8 Å, wide peaks could be observed in g(r)_O–H_-r and g(r) _N–H_-r, which indicated a strong Van der Waals’ forces and the electrostatic interactions among CL-20 and *p*-xylene.

## 4. Experimental Section

### 4.1. Crystallization of CL-20 p-Xylene Solvate by Evaporation

A glass vial was loaded with γ-CL-20 (2.19 mg, 5.0 µmol) and *p*-xylene (20 mL). Mild heating was utilized to completely dissolve all solids. About 20 mL of the solution was prepared and then carefully filtered using 0.45 µm PTFE filter into a clean glass vial. The solution was allowed to evaporate at room temperature over several days, single crystals were obtained and collected and then used for further characterization.

### 4.2. Characterization

Crystals suitable for X-ray crystallographic analysis were obtained as mentioned above. The CL-20 *p*-xylene solvate crystal structure was determined by a Rigaku RAXIS IP diffractometer and SHELXTL crystallographic software package of molecular structure. The single crystals were mounted on a Rigaku RAXIS RAPID IP diffractometer equipped with a graphite-monochromatized MoKα radiation (λ = 0.71073 Å). Data were collected by the ω scan technique. The structure was solved by direct method with SHELXS-97 [43] and expanded by using the Fourier technique. The non-hydrogen atoms were refined anisotropically. The hydrogen atom was determined with theoretical calculations and refined with an isotropic vibration factor.

Simultaneous TG-DSC of crystal was carried out on a thermal analysis system (Netzsch STA-449F3, Selb, Germany). The thermal behavior of sample was studied over a range at a heating rate 10 °C/min from 50 °C to 500 °C in nitrogen atmosphere.

FTIR spectra were recorded on a Bruker tensor 27 instrument with KBr pellets. The resolution was 4 cm^−1^, and the scan range was 400–4000 cm^−1^. The sample was ground with KBr powder and then pressed to obtain a suitably sized pellet for spectrum recording.

## 5. Conclusions

The CL-20 *p*-xylene solvate crystal was obtained through slow evaporation. The *p*-xylene molecules could enter the CL-20 crystal lattice by forming weak intermolecular interactions and stacking interactions, which affected not only the crystal structure but also the crystal morphology. The solvent effects on different crystal faces varied due to the diversity of functional groups, and then to change the crystal morphology significantly. With the modified AE, (002) and (11−1) faces were not visible at all due to the exposure of the less polar groups or atoms and hence a small solvent interaction, while the area percentage of (011) face was increased up to 12.51% in *p*-xylene solution from 7.81% in vacuum due to the strong solvent interaction. The predicted morphology was in good agreement with the observed experimental morphology, and this consistency validated the applicability of the modified AE model. Furthermore, the RDF analysis indicated that the *p*-xylene solvent molecules were adsorbed on the CL-20 faces mainly via interactions between hydrogen bonds, Van der Waals forces and electrostatic interactions. The molecular simulation could be an effective tool for the solvent selection to modify the morphology of the solvated CL-20 crystal and other solvated explosive crystals. In addition, molecular simulation could reduce the development cycle and the cost of explosive crystallization process.
